# Mesenchymal stromal cell therapy compared to SGLT2-inhibitors and usual care in treating diabetic kidney disease: A cost-effectiveness analysis

**DOI:** 10.1371/journal.pone.0274136

**Published:** 2022-11-04

**Authors:** Luke E. Barry, Grainne E. Crealey, Paul Cockwell, Stephen J. Elliman, Matthew D. Griffin, Alexander P. Maxwell, Timothy O’Brien, Norberto Perico, Ciaran O’Neill

**Affiliations:** 1 Centre for Public Health, Queen’s University Belfast, Belfast, United Kingdom; 2 John E. Cairnes School of Business and Economics, National University of Ireland, Galway (NUIG), Galway, Ireland; 3 Queen Elizabeth Hospital, University Hospitals Birmingham and Institute of Inflammation and Ageing, University of Birmingham, Birmingham, United Kingdom; 4 Orbsen Therapeutics Ltd., Galway, Ireland; 5 Regenerative Medicine Institute (REMEDI) at CÚRAM SFI Centre For Research in Medical Devices, School of Medicine, National University of Ireland, Galway (NUIG), Galway, Ireland; 6 Istituto di Ricerche Farmacologiche Mario Negri IRCCS, Bergamo, Italy; Istituto Superiore di Sanità and St. Camillus International University of Health Sciences, ITALY

## Abstract

**Background and objectives:**

To simulate the cost-effectiveness of Mesenchymal Stromal Cell (MSC) therapy compared to sodium/glucose co-transporter 2 inhibitors (SGLT2i) or usual care (UC) in treating patients with Diabetic Kidney Disease (DKD).

**Design, setting, participants, and measurements:**

This Markov-chain Monte Carlo model adopted a societal perspective and simulated 10,000 patients with DKD eligible for MSC therapy alongside UC using a lifetime horizon. This cohort was compared with an SGLT2i alongside UC arm and a UC only arm. Model input data were extracted from the literature. A threshold of $47,000 per quality-adjusted life year and a discount rate of 3% were used. The primary outcome measure was incremental net monetary benefit (INMB). Sensitivity analysis was conducted to examine: parameter uncertainty; threshold effects regarding MSC effectiveness and cost; and INMB according to patient age (71 vs 40 years), sex, and jurisdiction (UK, Italy and Ireland).

**Results:**

While MSC was more cost-effective than UC, both the UC and MSC arms were dominated by SLGT2i. Relative to SGLT2i, the INMB’s for MSC and UC were -$4,158 and -$10,085 respectively indicating that SGLT2i, MSC and UC had a 64%, 34% and 1% probability of being cost-effective at the given threshold, respectively. This pattern was consistent across most scenarios; driven by the relatively low cost of SGLT2i and demonstrated class-effect in delaying kidney failure and all-cause mortality. When examining younger patients at baseline, SGLT2i was still the most cost-effective but MSC performed better against UC given the increased lifetime benefit from delaying progression to ESRD.

**Conclusions:**

The evidence base regarding the effectiveness of MSC therapy continues to evolve. The potential for these therapies to reverse kidney damage would see large improvements in their cost-effectiveness as would targeting such therapies at younger patients and/or those for whom SGLT2i is contra-indicated.

## Introduction

According to the International Diabetic Federation, the worldwide prevalence of diabetes in 2019 was 463 million and is set to rise to 700 million by 2045 [1]. It carries a significant human cost in terms of morbidity, mortality, treatment costs and lost output for society. Global health expenditure on diabetes is estimated at USD$760 billion with these direct costs expected to reach $845 billion by 2045 [1]. Chronic kidney disease (CKD) is a common and major complication of Type 2 Diabetes Mellitus (T2DM).

CKD impacts negatively on survival and health-related quality of life (HRQoL) [2, 3]. The economic cost of CKD (including progression to end-stage renal disease [ESRD]) amongst individuals with diabetes is estimated to consume 13% of the US healthcare budget (Medicare), with an annual cost ranging from USD$20,000 (for a patient with CKD stage 2 (CKD2) attributed to Diabetic Kidney Disease [DKD]) to USD$80,000 (for a patient with ESRD requiring dialysis) [4]. Costs rise at an increasing rate in later CKD stages, in part due to increasing inpatient care [5].

Currently, no treatments are available to restore kidney function in T2DM patients with CKD. Current therapy is limited to pharmacological and lifestyle interventions aimed at delaying further kidney damage. New treatments (cell-based therapy and biological agents) for delaying disease progression show promise [6]. Mesenchymal stromal cells (MSC) may be effective in repairing tissue after injury to the kidneys [7, 8]. If MSC and other regenerative therapies can delay progression to ESRD, there would be significant health and, possibly, economic gains. Achieving this is dependent on identification of suitable patients earlier in the disease pathway to allow timely initiation of therapy: specifically younger patients and/or those in the early stages of DKD [7]. Research suggests these therapies may be cost-effective relative to usual care (UC) however, the cost at which they can be produced plays an important role in their viability [9].

Glucose-lowering agents have also recently demonstrated a reno-protective effect in patients with DKD beyond the control of hyperglycaemia [10]. The use of sodium/glucose co-transporter 2 inhibitors (SGLT2i) has demonstrated success in delaying onset of ESRD among patients with DKD and reducing mortality [11–13]. They are likely to be more cost-effective than UC [14, 15]. With efficacy data now available on the use of stem cell therapies in treating patients with DKD [16], an opportunity exists to examine the potential cost-effectiveness of these therapies compared to UC as well as to UC+SGLT2i.

This paper presents the results of a cost-effectiveness analysis using a Markov cohort model to compare MSC therapy alongside UC, SGLT2i alongside UC and UC alone in delaying onset of ESRD and death.

## Research design and methods

### Model structure and perspective

**Fig 1:**
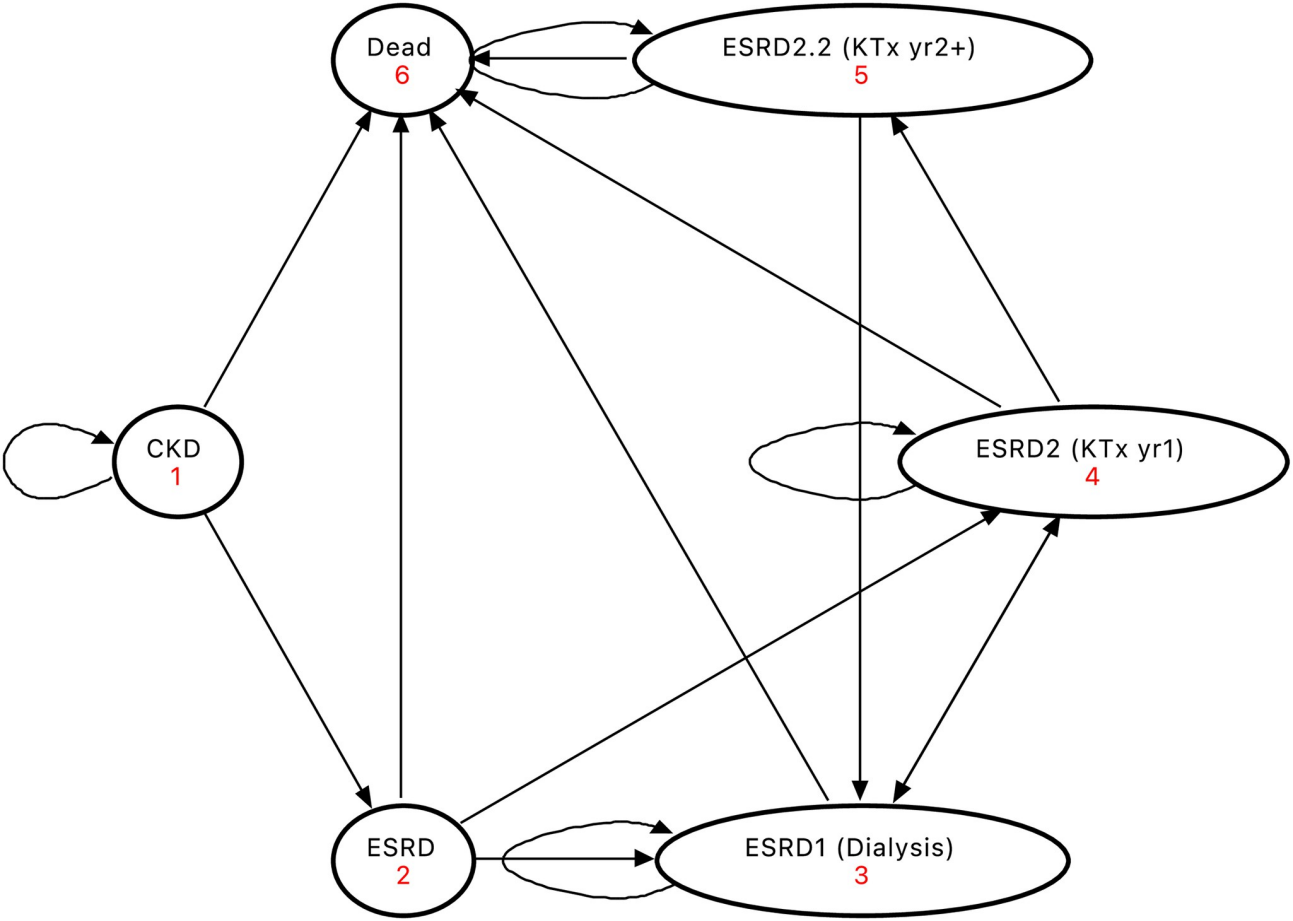
Markov model diagram. ESRD1 refers to Dialysis and ESRD2 refers to Kidney Transplant (KTx); ESRD2 is divided into year 1 (ESRD2) and subsequent years (ESRD2.2) to account for reduced costs after first post-transplant year. CKD—Chronic Kidney Disease; ESRD–End Stage Renal Disease; KTx–Kidney Transplant.

A Markov model, following a similar structure to other models analysing CKD [17], was developed with input from senior clinical specialists. Reporting conforms with the Consolidated Health Economic Evaluation Reporting Standards (CHEERS) 2022 [18] (S1 Table in S1 File). The model was constructed using CKD stages categorised according to estimated Glomerular Filtration Rate (eGFR) [19]. Individuals with CKD3 or CKD4 (eGFR range 15-59mL/min/1.73m^2^) can progress to ESRD, defined as CKD5 (eGFR<15mL/min/1.73m^2^) or to the terminal state (Dead) according to the monthly transition probabilities of disease progression (Fig 1). Individuals with ESRD may be offered one of two types of renal replacement therapy (RRT); dialysis or kidney transplantation. Two stages were included for kidney transplantation to allow for reduced costs after the first post-transplant year. The base-case scenario analysed patients with a starting age of 71 years, eGFR = 35mL/min/1.73m^2^, ACR = 403mg/g, average sex and jurisdiction (S2 Table in S1 File).

Three strategies were compared: a UC strategy (excluding SGLT2i) based on costs, utilities and transition probabilities collected from the literature, as well as MSC and SGLT2i arms each including the treatment costs for these therapies alongside UC and the expected changes in the probability of transitioning to ESRD or death associated with treatment. Individuals receiving MSC therapy are likely to receive treatment in CKD3 or CKD4 [16] and, where data were available, input parameters for those with CKD used combined CKD3 and CKD4 estimates, otherwise those for CKD4 only are used. While fluctuations in GFR may occur [20] it was assumed that the progressive nature of the disease was such that recovery, with sustained improvements in GFR, was unlikely. Or in the case of MSC therapy, that there was not yet sufficient evidence to justify this scenario. Therefore, we assumed that a patient would not remit to previous states. A detailed methodology outlining the identification and implementation of costs, utilities, transition probabilities along with model validation is in the S1 Supporting Information File. This study was deemed exempt from ethics approval by Queen’s University Belfast Faculty Research Ethics Committee/Institutional Review Board because it used anonymised data.

Arms were compared using the incremental cost-effectiveness ratio (ICER) and incremental net monetary benefit (INMB). A societal perspective was adopted, as advocated in economic evaluations [21], and results were examined across three jurisdictions: Ireland, Italy and the UK. These jurisdictions were chosen as they had contributed data to a trial assessing the safety and efficacy of a novel MSC therapy (the Novel Stromal Cell Therapy for Diabetic Kidney Disease Study; NCT 02585622). Different discount rates are recommended in different countries [22]; Italy (3%), Ireland (4%) and UK (3.5%). Half-cycle corrections were applied as part of the discounting of costs and benefits and, unless examining individual jurisdictions, we applied a conservative discount rate of 3%. For a more consistent comparison of results across models, all costs were converted to USD 2019 and a conservative Willingness-to-Pay (WTP) threshold per Quality-Adjusted Life Year (QALY) was estimated as the OECD average Gross Domestic Product (GDP) per capita (USD$47,000) [23–25]. One cycle in the model represents one month and all inputs into the model, where estimated annually, were converted to monthly data [26]. The model was run for 100 years or until everyone had died and was constructed in TreeAge Pro Healthcare 2020 [27].

### Sensitivity analysis

One-way sensitivity analysis examined changes in cost-effectiveness rankings according to a range of scenarios. Changes to the base-case scenario were examined by modelling: a younger cohort of patients at baseline (40 years)—reflective of the lower bound for ongoing enrolment of patients into an MSC trial (NCT 02585622); males and females separately; and jurisdictions separately (UK, Ireland and Italy). A probabilistic sensitivity analysis (PSA) was conducted to estimate the probability of each therapy being cost-effective across WTP thresholds by simulating a cohort of patients 10,000 times and summarising results with costs, utilities and transition probabilities drawn from their assumed distributions (S2-S8 Tables in S1 File) [26, 28].

### Threshold analysis

Given the uncertainty associated with MSC treatment, a two-way threshold analysis was conducted varying MSC effect and cost simultaneously to highlight their trade-off in establishing the point of indifference between MSC therapy and the dominant strategy. This was estimated separately for those aged 71 and 40 years at baseline.

## Results

The base-case scenario focused on the societal perspective (i.e. healthcare costs and lost productivity), using average values across jurisdictions and sex, a starting age of 71 years, ACR = 403mg/g, and eGFR = 35mL/min/1.73m^2^. SGLT2i dominated both UC and MSC therapy as it was the least costly and most effective. The UC arm had 6.28 QALYs at a cost of $159,978 per patient, the MSC arm generated 6.39 QALYs at $158,770 per patient, and the SGLT2i arm generated 6.46 QALYs at $158,131 per patient (Table 1).

**Table 1:** Cost-effectiveness analysis results across scenarios.

Strategy/ Scenario	Costs (USD)	QALYs	Comparison	Incremental Cost (USD)	Incremental QALY	ICER (USD/QALY)	INMB ($47k/QALY)	Dominance
**Base-case scenario**								
**SGLT2i**	158131	6.46						undominated
**MSC**	158770	6.39	MSC vs. SGLT2i	639	-0.07	-8538	-4158	abs. dominated
**Usual Care**	159978	6.28	UC vs SGLT2i	1847	-0.18	-10534	-10085	abs. dominated
**Baseline age: 40 years**								
**SGLT2i**	1909729	29.95						undominated
**MSC**	1948430	29.45	MSC vs. SGLT2i	38701	-0.50	-77631	-62132	abs. dominated
**Usual Care**	1997307	28.93	UC vs. SGLT2i	87578	-1.02	-86011	-135435	abs. dominated
**Country: UK**								
**SGLT2i**	150957	6.24						undominated
**MSC**	152407	6.17	MSC vs. SGLT2i	1450	-0.07	-20250	-4814	abs. dominated
**Usual Care**	153295	6.08	UC vs. SGLT2i	2337	-0.17	-13938	-10218	abs. dominated
**Country: Ireland**								
**SGLT2i**	150777	6.26						undominated
**MSC**	152558	6.18	MSC vs. SGLT2i	1782	-0.07	-25207	-5104	abs. dominated
**Usual Care**	153479	6.09	UC vs. SGLT2i	2703	-0.17	-16317	-10487	abs. dominated
**Country: Italy**								
**MSC**	172991	6.84						undominated
**SGLT2i**	174460	6.93	SGLT2i vs MSC	1469	0.08	17674	2438	undominated
**Usual Care**	174911	6.73	UC vs. MSC	1920	-0.11	-17268	-7145	abs. dominated
**Sex: Female**								
**SGLT2i**	172109	7.00						undominated
**MSC**	172940	6.93	MSC vs. SGLT2i	832	-0.07	-12034	-4079	abs. dominated
**Usual Care**	175079	6.84	UC vs. SGLT2i	2971	-0.16	-18326	-10590	abs. dominated
**Sex: Male**								
**SGLT2i**	144268	5.93						undominated
**MSC**	144779	5.85	MSC vs. SGLT2i	511	-0.08	-6601	-4148	abs. dominated
**Usual Care**	145167	5.75	UC vs. SGLT2i	898	-0.18	-4966	-9401	abs. dominated

Scenario analysis (Table 1) showed that the pattern of SGLT2i dominating both UC and MSC therapy was consistent when examining individuals with a younger starting age (40 years), individual jurisdictions (except Italy where the cost of SGLT2i was relatively higher) and males and females separately. Across scenarios, MSC was usually dominated by SGLT2i but was consistently more effective and less costly than UC.

**Fig 2:**
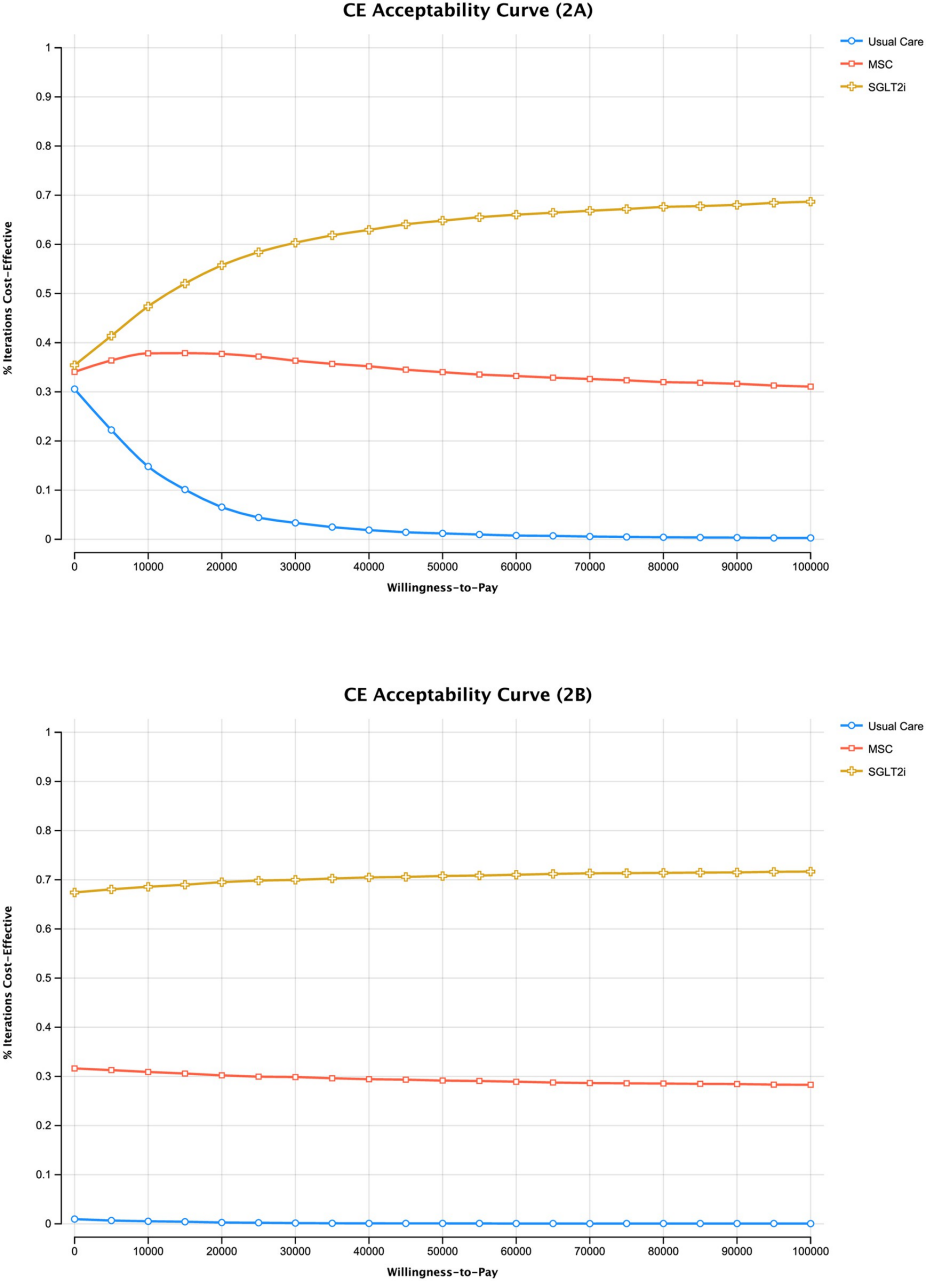
Cost-effectiveness acceptability curve displaying the probability of each treatment (SGLT2i, MSC, Usual Care) being cost-effective at different willingness-to-pay thresholds for those aged 71 years at baseline (2A) and those aged 40 (2B).

The cost-effectiveness acceptability curve shows the probability of each therapy being cost-effective across WTP thresholds. Fig 2A presents the base-case scenario and shows that, across thresholds, SGLT2i was most likely to be cost-effective. At a threshold of $47,000/QALY, SGLT2i had a 64% chance of being cost-effective followed by MSC (34%) and UC (1%). When using a starting age of 40 years (Fig 2B), the same pattern held; SGLT2i had a 71% probability of being cost-effective, followed by MSC (29%) and UC (<0.1%).

**Fig 3:**
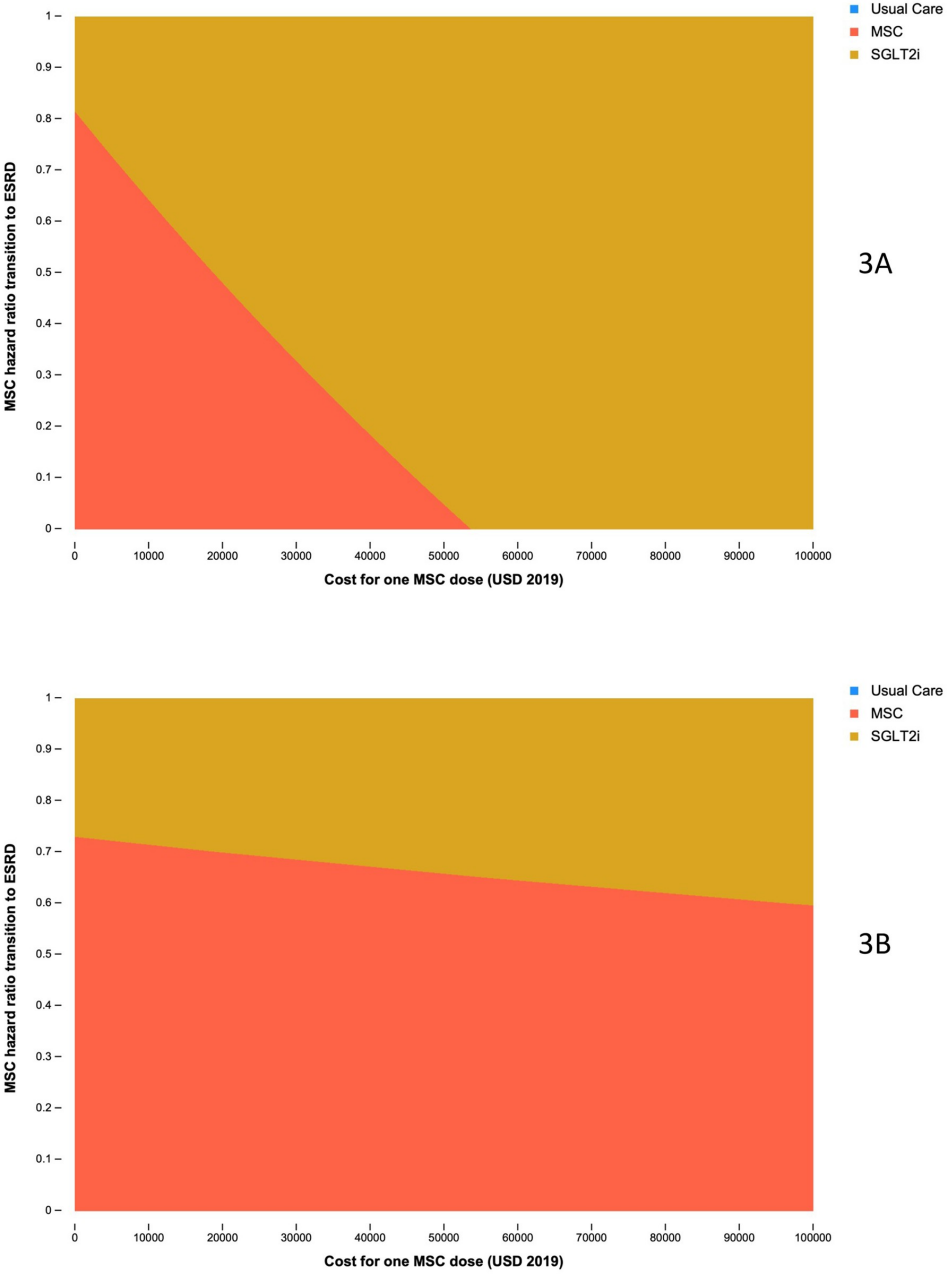
Two-way threshold analysis examining the incremental net monetary benefit according to variation in MSC treatment cost ($/dose) and effect using an ICER threshold of USD $47,000 for those aged 71 years at baseline (3A) and those aged 40 (3B). Usual Care is represented by blue in Fig 3A & 3B. That no blue is visible highlights the fact that it is always dominated by either MSC or SGLT2i at the given WTP threshold.

Two-way threshold analysis was used to examine the trade-off between expected cost and effect in determining the INMB using a WTP threshold of $47,000/QALY according to different baseline ages of 71 (Fig 3A) and 40 years (Fig 3B). The downward sloping line between the yellow and red regions highlights the negative trade-off between MSC cost and effect. A lower HR, indicating a stronger treatment effect, implies that at higher treatment costs MSC therapy may be more cost-effective than UC and SGLT2i. The red region shows the area at which MSC therapy was more cost-effective than UC and SGLT2i and in all other cases SGLT2i was most cost-effective (yellow region).

MSC therapy was unlikely to be more cost-effective than SGLT2i if the HR for transition to ESRD was greater than approximately 0.8 for those aged 71 (Fig 3A) albeit reflecting the unrealistic scenario where MSC treatment cost was $0/dose. For lower HRs, greater flexibility in treatment cost was observed up to a maximum of approximately $53,000/dose (in the scenario where MSC therapy halted progression to ESRD [HR = 0]). Greater flexibility was observed when targeting MSC therapy at those aged 40 (Fig 3B); Modest reductions from a HR below 0.72 suggest that treatment was still likely to be more cost-effective than UC and SGLT2i even for high MSC therapy costs.

## Discussion

This study provides a three-arm comparison examining the cost-effectiveness of MSC therapy alongside UC among patients with DKD in comparison to a ‘UC only’ arm and an ‘SGLT2i+UC’ arm. The results of the base-case scenario show that, relative to the UC and MSC arms, treatment with SGLT2i alongside UC was cost-saving, resulted in more QALYs and supports recent inclusions of SGLT2i as part of UC [29]. This was driven by the relatively low drug cost and the demonstrated class-effect in delaying kidney failure and death [11–13]. This scenario modelled patient characteristics according to those of individuals currently enrolled in clinical trials for a cell-based therapy in treating DKD [16]. Further scenario analysis demonstrated that this result held when considering: younger patients at baseline (age 40); males and females separately; and in two of three jurisdictions (UK and Ireland) examined.

A recent systematic review of economic evaluations of SGLT2i also supported the conclusion that treatment with SGLT2i is more cost-effective than UC [15]. Economic evaluations of cell-based therapy in treating DKD are sparse though De Vries et al. [9] modelled a scenario of a one-time, cell-based therapy compared to UC. They found that it was likely to be cost-effective (at a WTP threshold ranging from €20,000–40,000) if it delayed entry to ESRD by between 0.2–0.5 years relative to an average duration in ESRD of 2 years, i.e. a 10%-25% increase in time spent in CKD4. In our model, the median time spent in the CKD stage in the UC arm was 5.5 years and 5.7 years in the MSC arm. The difference (0.2) corresponds to the lower bound of the De Vries estimate, and results in a modest proportionate increase in time spent in the CKD3/4 stage of 4% from our estimated HR of 0.83. Relative to UC, MSC was likely to be cost-effective at a WTP threshold of $47,000. That we conducted a PSA and used efficacy data from a published trial suggests our results may provide a more robust modelling of the potential cost-effectiveness of single-dose MSC for DKD. Furthermore, we compared UC with both cell-based and SGLT2i therapies, demonstrating that MSC therapy, as well as UC, was also dominated by SGLT2i. The introduction of new pharmacological agents such as SGLT2i raises the bar for feasibility of complex/advanced therapies to impact clinical practice. However, subsequent analyses highlighted scenarios under which novel stem cell therapies may be more feasible in treating DKD and helps to guide future research.

PSA demonstrated that, while MSC therapy was likely to be cost-effective relative to UC at a WTP threshold of $47,000, SGLT2i dominated both of these with a 64% probability of being cost-effective. However, the novelty of MSC therapies in treating DKD means that there is high uncertainty as to the expected cost and effect in terms of delaying kidney failure or indeed potentially reversing kidney injury [7]. In the UK, NICE recommends examination of cost-effectiveness results according to biologically plausible sub-groups [30]. Given the potential lifetime benefit from treating younger patients, in terms of both increased HRQoL and labour productivity, we considered additional scenarios examining younger individuals at baseline.

An earlier pilot study of MSC therapy involved a younger cohort of patients than those used in our base-case analysis [31] and an ongoing trial used a lower bound for patient enrolment of 40 years (NCT 02585622). When using a baseline age of 40 years we found that, compared to SGLT2i and MSC, UC was even less likely to be cost-effective (<0.1% at age 40 vs 1% at age 71) at the $47,000 WTP threshold. Although SGLT2i still dominated both MSC and UC, potential adverse events related to SGLT2i, like diabetic ketoacidosis or genitourinary infections [32, 33], could create preferential conditions for MSC therapy over UC especially for younger patients, those who are at increased risk of diabetic ketoacidosis, and those with a GFR<60mL/min/1.73m^2^ [34]. Continued efforts to identify younger individuals at higher risk of developing DKD or in earlier stages of this disease, through biomarkers for example [8], or individuals for whom SGLT2i are contra-indicated may highlight specific patient groups for whom these MSC-based therapies are cost-effective. That cost-effectiveness varied between jurisdictions related to the cost of SGLT2i, also points to scenarios in which MSC may be preferred.

It will be important to provide updated cost-effectiveness analyses as more data becomes available on the effectiveness of MSC therapy in treating DKD. Currently however, Fig 3A and 3B offer a guide as to the likely cost-effectiveness of MSC therapy at varying combinations of cost and effect in delaying onset of ESRD. This threshold analysis shows that, should improvements in efficacy be realised from further trials (i.e. reduced HR for transitioning to ESRD), this is likely to impact the cost-effectiveness of MSC therapy relative to UC and SGLT2i, especially for younger patients (Fig 3B). This may justify the typically high price of these therapies [35] and, although focusing on a different disease, is consistent with other economic evaluations of MSC therapies [36]. The potential success of MSC therapies in treating DKD is, therefore, partially dependent on their effectiveness in delaying transition to ESRD, their price relative to other therapies and the age at which individuals are treated. Considerable uncertainty exists around these parameters. Should MSC prove effective and production move to scale, not only would the HR for progression fall, but so too would its cost, assuming even modest economies of scale in production are achievable.

The results presented here may also act as a guide for ongoing enrolment in trials of cell and regenerative therapies and for the value of ascertaining improved efficacy data in younger individuals for whom the lifetime benefit of intervention is greatest. Currently, SGLT2i therapy provides a cost-effective option compared to MSC therapy and UC across all ages. In the US, the Food and Drug Administration (FDA) and the National Institutes for Health (NIH) have highlighted the use of susceptibility/risk biomarkers as being of value in guiding preventative strategies [37]. Biomarkers such as eGFR decline, which represent credible surrogate endpoints [38], offer promise as screening tools for earlier identification of individuals at risk of longer-term negative health outcomes and/or likely to respond to MSC therapy [6].

### Limitations

Firstly, UC was not clearly defined as we collated estimates of DKD costs and utilities across a number of studies. However, this should also mitigate against aberrations from a single study while the restrictions applied in extracting estimates also improves their robustness. For example, we included only total healthcare costs and estimates including only inpatient costs were excluded or we used utilities almost entirely from the same HRQoL measure—the EQ5D3L. The random effects meta-analysis conducted also accounts for potential differences in average estimates of cost or utility between studies. Where possible, estimates relating specifically to CKD among individuals with T2DM were used. However estimates relating more broadly to CKD or to other CKD aetiologies such polycystic kidney disease were also used—for example, labour productivity [39]. As we are focused on relative values between arms and disease states, we expect that the results presented here provide a realistic approximation of the cost-effectiveness of these therapies among T2DM patients with CKD. That our results align with a systematic review of SGLT2i economic evaluations provides reassurance as to their validity [15]. Finally, as with any modelling exercise, we were not able to consider all possible outcomes from therapy. There may be additional benefits from SGLT2i therapy (or MSC) in terms of reduced cardiovascular disease risk and body weight or adverse events like diabetic ketoacidosis or genitourinary infections [29, 32, 33] which were not in the model. Additionally, we estimated the effect of MSC therapy in delaying entry to ESRD based on trial data from a similar cell-based therapy. However, MSC therapies may have regenerative effect on kidneys [7]. This would likely improve the cost-effectiveness of MSC relative to other therapies via a reduced HR of transitioning to ESRD among MSC-treated patients.

## Conclusion

We found that SGLT2i alongside UC dominated both MSC therapy alongside UC and UC alone in treating patient with DKD. This was driven by the relatively low drug cost and the demonstrated class-effect of SGLT2i in delaying kidney failure and all-cause mortality [11–13]. There remains high uncertainty in the evidence base for MSC therapies in treating DKD. Threshold analysis suggested that modest increases in their effectiveness in delaying transition to ESRD could justify their higher cost relative to SGLT2i. The potential for these therapies to reverse kidney damage would see further improvements in their cost-effectiveness as may targeting such therapies at patients for whom SGLT2i may be contra-indicated and/or younger populations for whom the lifetime benefit is greatest. Given the lack of available published data on the effectiveness of such therapies, additional trials with larger sample sizes, especially those including younger individuals with DKD, provide a valuable avenue for collecting such data.

## Supporting information

S1 File (DOCX)
