# Supplementary material for: Mesenchymal stromal cell therapy compared to SGLT2-inhibitors and usual care in treating diabetic kidney disease: A cost-effectiveness analysis
Source: PLoS One. 2022 Nov 4;17(11):e0274136. doi: 10.1371/journal.pone.0274136 (PMC9635741; doi:10.1371/journal.pone.0274136)
Supplement: S1 File — (DOCX) [file pone.0274136.s001.docx]

# S1 File

# S1 Table: CHEERS 2022 Checklist

| **Topic** | **No.** | **Item** | **Location where item is reported** |
| --- | --- | --- | --- |
| **Title** |  |  |  |
|  | 1 | Identify the study as an economic evaluation and specify the interventions being compared. | Manuscript p.1 |
| **Abstract** |  |  |  |
|  | 2 | Provide a structured summary that highlights context, key methods, results, and alternative analyses. | Manuscript p.2 |
| **Introduction** |  |  |  |
| **Background and objectives** | 3 | Give the context for the study, the study question, and its practical relevance for decision making in policy or practice. | Manuscript p.4-5 |
| **Methods** |  |  |  |
| **Health economic analysis plan** | 4 | Indicate whether a health economic analysis plan was developed and where available. | A report was developed in consultation with the clinical team but was not published (available upon request) |
| **Study population** | 5 | Describe characteristics of the study population (such as age range, demographics, socioeconomic, or clinical characteristics). | Manuscript p.6 and the supplement Table S4 |
| **Setting and location** | 6 | Provide relevant contextual information that may influence findings. | Manuscript p.6-7 |
| **Comparators** | 7 | Describe the interventions or strategies being compared and why chosen. | Manuscript p.6 |
| **Perspective** | 8 | State the perspective(s) adopted by the study and why chosen. | Manuscript p.7 |
| **Time horizon** | 9 | State the time horizon for the study and why appropriate. | Manuscript p.7 |
| **Discount rate** | 10 | Report the discount rate(s) and reason chosen. | Manuscript p.7 |
| **Selection of outcomes** | 11 | Describe what outcomes were used as the measure(s) of benefit(s) and harm(s). | Manuscript p.6-7 |
| **Measurement of outcomes** | 12 | Describe how outcomes used to capture benefit(s) and harm(s) were measured. | Manuscript p.6 and supplement p.2-10 |
| **Valuation of outcomes** | 13 | Describe the population and methods used to measure and value outcomes. | Supplement p.2-10 |
| **Measurement and valuation of resources and costs** | 14 | Describe how costs were valued. | Supplement p.2-10 |
| **Currency, price date, and conversion** | 15 | Report the dates of the estimated resource quantities and unit costs, plus the currency and year of conversion. | Manuscript p.7 and Supplement p.1 |
| **Rationale and description of model** | 16 | If modelling is used, describe in detail and why used. Report if the model is publicly available and where it can be accessed. | Manuscript p.5-7 (The model is not publicly available) |
| **Analytics and assumptions** | 17 | Describe any methods for analysing or statistically transforming data, any extrapolation methods, and approaches for validating any model used. | Supplement p.5-7 & p.10-11 |
| **Characterising heterogeneity** | 18 | Describe any methods used for estimating how the results of the study vary for subgroups. | Manuscript p.7 |
| **Characterising distributional effects** | 19 | Describe how impacts are distributed across different individuals or adjustments made to reflect priority populations. | Manuscript p.7 |
| **Characterising uncertainty** | 20 | Describe methods to characterise any sources of uncertainty in the analysis. | Manuscript p.7-8 and Supplement p.1-2 |
| **Approach to engagement with patients and others affected by the study** | 21 | Describe any approaches to engage patients or service recipients, the general public, communities, or stakeholders (such as clinicians or payers) in the design of the study. | Manuscript p.5 (Model was developed with input from senior clinical specialists) |
| **Results** |  |  |  |
| **Study parameters** | 22 | Report all analytic inputs (such as values, ranges, references) including uncertainty or distributional assumptions. | Supplement p.2-10 |
| **Summary of main results** | 23 | Report the mean values for the main categories of costs and outcomes of interest and summarise them in the most appropriate overall measure. | Manuscript p.8 |
| **Effect of uncertainty** | 24 | Describe how uncertainty about analytic judgments, inputs, or projections affect findings. Report the effect of choice of discount rate and time horizon, if applicable. | Manuscript p.8-9 |
| **Effect of engagement with patients and others affected by the study** | 25 | Report on any difference patient/service recipient, general public, community, or stakeholder involvement made to the approach or findings of the study | Manuscript p.12 (Results helping to guide patient enrolment for MSc trials) |
| **Discussion** |  |  |  |
| **Study findings, limitations, generalisability, and current knowledge** | 26 | Report key findings, limitations, ethical or equity considerations not captured, and how these could affect patients, policy, or practice. | Manuscript p.10-13 |
| **Other relevant information** |  |  |  |
| **Source of funding** | 27 | Describe how the study was funded and any role of the funder in the identification, design, conduct, and reporting of the analysis | Manuscript p.15 |
| **Conflicts of interest** | 28 | Report authors conflicts of interest according to journal or International Committee of Medical Journal Editors requirements. | Manuscript p.15 |

*From:* Husereau D, Drummond M, Augustovski F, et al. Consolidated Health Economic Evaluation Reporting Standards 2022 (CHEERS 2022) Explanation and Elaboration: A Report of the ISPOR CHEERS II Good Practices Task Force. Value Health 2022;25. <doi:10.1016/j.jval.2021.10.008>

## Methods

#### Transition Probabilities between disease states

Annual transition probabilities of progressing through CKD stages were extracted from the literature (1-3). Specifically, the monthly probability of entering ESRD from CKD3/4 was estimated using the 4-variable/5-year risk equation for non-North American countries (2; 3). These required baseline estimates of age, sex, Albumin/Creatinine Ratio (ACR; mg/g) and eGFR (mL/min/1.73m^2^) which were estimated as the average across arms in (4) as these reflect the characteristics of individuals eligible for MSC therapy (S2 Table). Upon entry to ESRD, patients are modelled separately according to the treatment they receive whether dialysis or transplantation, however individuals on dialysis may subsequently receive a transplant while individuals who receive a transplant may also receive dialysis, for example if the transplant is unsuccessful or an individual requires another transplant later in life. Estimates of annual transition probabilities between dialysis and transplant were taken from (1).

To convert annual estimates into monthly estimates, these were first converted to rates before being adjusted to monthly rates then converted to monthly probabilities (5). Upper and lower ranges for the probabilities were identified and using the formula from (6), SEs were estimated for PSA according to the range and the number of study estimates (see the worked example below). The probability of initially receiving either a kidney transplant or dialysis following transition to ESRD (CKD5) was estimated as an age-dependent distribution from a Dutch national registry with younger individuals more likely to receive a transplant and older individuals more likely to receive dialysis (7). To account for the progressive nature of the disease, a Markov jump process was included (see ‘Validation’ section below) with the baseline probability of transferring between states an increasing function of the number of cycles (5). S2 Table sets out the transition probabilities and related parameters for estimating transitions across disease stages based on the analysis described.

S2 Table: Annual transition probabilities and baseline patient characteristics associated with ESRD

| Name | Description | Value | Standard Error | Source |
| --- | --- | --- | --- | --- |
| _init_age | Initial age (years) of individuals/cohort in the model | 71 | 1.51 | (4), estimated across arms |
| _sex | Distribution of males to females | 0.8 | Alpha: 24  Beta: 6 | (4), estimated across arms |
| eGFR | Baseline eGFR (mL/min/1.73m^2^) of individuals/cohort in the model | 34.9 | 1.9 | (4), estimated across arms |
| ACR | Baseline ACR (mg/g) of individuals/cohort in the model | 403 | 122 | (4), estimated across arms |
| tp_E1E2 | Annual probability of transition from Dialysis to Transplant | 0.06 | 0.049 | (1) |
| tp_E2E1 | Annual probability of transition from Transplant to Dialysis | 0.08 | 0.046 | (1) |

###### Converting annual probabilities to monthly probabilities – Worked example

Below sets out this process for estimating the SE for the annual probability of transitioning from Dialysis to Transplant and the conversion of this annual probability to a monthly transition probability (TPr). This value of 0.055 was taken from Surgue et al 2019 (1), was based on nine studies and had a minimum of 0.005 and a maximum of 0.15. The conversion factor using the maximum and minimum values to an SE for an estimate based on nine studies was 0.337 (6).

SE = (0.15-0.005)*0.337 = 0.049

Monthly TPr = 1-exp(-(-(LN(1-0.055))/12)) = 0.0047

#### Healthcare costs and Utilities

For healthcare costs and utilities, estimates from the literature were identified for CKD stages and ESRD as well as measures of dispersion for the Probabilistic Sensitivity Analysis (PSA). To allow for the inclusion of indirect as well as direct healthcare costs associated with disease progression, data collected on healthcare costs were restricted to studies which estimated all healthcare costs not just those related to a specific type of healthcare utilisation. Where a study reported costs for combined CKD stages (e.g. healthcare costs for CKD stage 3 and 4 together), this was input as the cost for CKD 3 and CKD 4. Costs for the two different types of dialysis (haemodialysis and peritoneal dialysis) were combined or, where a single study reported both, a weighted average was estimated according to sample sizes within each study. Only utilities estimated using EQ5D3L were included except for one use of EQ5D5L for kidney transplant in the UK as this was very similar to EQ5D3L estimates for the same disease stage (8). Unless costs were already provided in 2019 USD, costs were converted to USD based on purchasing power parity estimates for different countries inflated to 2019 estimates using Purchasing Power Parity conversion rates from the International Monetary Fund through a tool developed with support from the Cochrane group (<https://eppi.ioe.ac.uk/costconversion/default.aspx>).

Meta-analysis was used to estimate average healthcare costs for CKD stages and ESRD with Standard Errors (SE) across studies using a random effects maximum likelihood model in Stata 16 (9) to facilitate PSA. Some studies did not provide a measure of dispersion, such as standard error (SE) or confidence interval. Rather than discard this estimate from the meta-analysis, the missing SEs were estimated as a linear function of the study mean utility or mean log of the cost, CKD stage and country using ordinary least squares (OLS) regression (10). As a robustness check to this approach, where measures of dispersion were not missing, we conducted a meta-analysis using the non-missing SEs and those predicted from the OLS model and the results were compared with the null hypothesis for differences between groups rejected (p < 0.001).

Cost and utility data were not consistently available across all CKD stages for UK, Ireland and Italy separately. The average healthcare costs and utilities, according to the random effects meta-analysis described above, were used for each disease stage across all countries. This avoided bias from any one study-/country-specific estimate of cost or utility for a single disease stage and helped to ensure face validity of costs and utilities across stages (i.e. greater cost and lower utility associated with more severe disease (11)). S3 and S4 Tables set out the mean and SE healthcare costs and utility values by disease stage based on the analysis described.

S3 Table: Annual treatment costs (USD 2019) associated with CKD and ESRD

| Name | Description | Value ($) | Standard Error | Source |
| --- | --- | --- | --- | --- |
| hc_CKD | Annual healthcare cost – CKD3/4 | 15,052 | 2,640 | (7; 11; 12) |
| hc_ESRD | Annual healthcare cost – ESRD/CKD5 | 28,599 | 7,974 | (7; 11; 12) |
| hc_ESRD1 | Annual healthcare cost - Dialysis | 77,626 | 5,795 | (7; 11; 12) |
| hc_ESRD2_1 | Annual healthcare cost - Transplant (year 1) | 40,074 | 5,439 | (7; 11; 12) |
| hc_ESRD2_2 | Annual healthcare cost - Transplant (year 2+) | 15,214 | 3,250 | (7; 11; 12) |

S4 Table: Utility during CKD, ESRD, ESRD1 (Dialysis) and ESRD2 (Transplant)

| Name | Description | Value | Standard Error | Source |
| --- | --- | --- | --- | --- |
| u_CKD | Annual utility – CKD3/4 | 0.81 | 0.014 | (7; 11; 13) |
| u_ESRD | Annual utility – ESRD/CKD5 | 0.76 | 0.018 | (7; 11; 13) |
| u_ESRD1 | Annual utility - Dialysis | 0.63 | 0.024 | (7; 11; 13) |
| u_ESRD2 | Annual utility - Transplant | 0.79 | 0.042 | (7; 11; 13) |
| u_Dead | Annual utility - Dead | 0 |  |  |

#### Societal Costs

The human capital approach was used to estimate lost productivity costs as part of the societal cost scenario as recommended by Health Technology Assessment regulators alongside healthcare costs (14; 15). These were estimated using average income, labour force productivity rates (LFPR) and pensionable age for the UK, Ireland and Italy separately from the OECD (16; 17). S5 Table sets out the lost productivity estimates and related parameters for estimating societal costs by disease stage based on the analysis described.

LFPR for CKD stages was only obtained for those on dialysis (18). To estimate LFPR for other CKD stages, we used data reporting lost productivity (LP) along with SEs from the Work Impairment and Activity Impairment (WPAI) general health questionnaire (19) across a number of European countries according to CKD stage (20). This data was for individuals with autosomal dominant polycystic kidney disease (ADPKD) rather than DKD, which is generally associated with greater comorbidity than ADPKD per CKD stage and would therefore likely incur greater productivity loss. However as here we are principally interested in the ratio of productivity loss between CKD stages, absolute levels of LP per stage are less relevant. Having the LFPR for dialysis as well as the LP for all CKD stages, including dialysis, allowed the rescaling of the LFPR for CKD stages according the ratio of LP for dialysis to the LP for the CKD stage of interest:

$${LFPR}_{CKD}=(1+\left( \frac{{{LP}_{dialysis}- LP}_{CKD}}{{LP}_{dialysis}} \right)){*LFPR}_{dialysis}$$

LP costs in the model were estimated as the difference between national LFPR, for Italy for example, and estimated LFPR for the relevant CKD stage. This was then multiplied by the average income for that country up to the corresponding pensionable age. For the average scenario across UK, Ireland and Italy, the arithmetic mean of the LFPR, income and pensionable ages were used.

S5 Table: Annual productivity costs (USD 2019) and relevant model parameters associated with CKD and ESRD

| Name | Description | Value | Standard Error | Source |
| --- | --- | --- | --- | --- |
| LFPR_Dialysis | Dialysis Labour Force Participation Rate | 26.2 | 6.7 | (18) |
| LFPR_Transplant | Transplant Labour Force Participation Rate | 42.1 |  | Estimated |
| LFPR_ESRD | ESRD/CKD5 Labour Force Participation Rate | 38.1 |  | Estimated |
| LFPR_CKD | CKD3/4 Labour Force Participation Rate | 46 |  | Estimated |
| LFPR_Ire | Ireland Labour Force Participation Rate | 62.1 | - | (16) |
| LFPR_Ita | Italy Labour Force Participation Rate | 49.9 | - | (16) |
| LFPR_UK | UK Labour Force Participation Rate | 63.5 | - | (16) |
| pa_Ire | Ireland pensionable age (years) | 66 | - | (17) |
| pa_Ita | Italy pensionable age (years) | 67 | - | (17) |
| pa_UK | UK pensionable age (years) | 64 | - | (17) |
| inc_Ire | Ireland average annual earnings (USD 2019) | 50,500 | - | (17) |
| inc_Ita | Italy average annual earnings (USD 2019) | 39,200 | - | (17) |
| inc_UK | UK average annual earnings (USD 2019) | 47,200 | - | (17) |
| pl_CKD | Productivity loss – CKD3/4 | 10.2 | 1.77 | (20), estimated across CKD stages |
| pl_ESRD | Productivity loss – ESRD/CKD5 | 22.8 | 3.94 | (20) |
| pl_ESRD1 | Productivity loss - Dialysis | 41.8 | 4.44 | (20) |
| pl_ESRD2 | Productivity loss - Transplant | 16.4 | 2.91 | (20) |

#### Mortality

All-cause mortality Hazard Ratios (HR) were extracted from the literature along with SEs (21; 22). While it is possible to consider death from specific causes such as cardiovascular disease, the use of separate death states for DKD and other causes was thought to unnecessarily complicate the model’s structure and, therefore, death from any-cause was used as the only terminal state. HRs estimating mortality in CKD were available for males and females corresponding to baseline eGFR and ACR, while HRs for dialysis or transplant were not sex-specific. Where the HR for CKD 3 was split into 2 stages (3a and 3b), only CKD 3b was used and combined with CKD 4 to estimate the HR for CKD and an average between males and females was also estimated (10). As the probability of dying in any stage is age-dependent, the most recent age- and sex-specific mortality tables for each country (UK [2016-2018], Ireland [2015-2017], and Italy [2015-2017]) were taken from the Human Mortality Database (23). These population mortality rates were multiplied by the HRs for the corresponding stage in the model and then converted to monthly transition probabilities using (5). S6 Table sets out the all-cause mortality HRs to be applied to the general population mortality rates and related parameters for estimating transitions to death across disease stages based on the analysis described.

S6 Table: All-cause mortality hazard ratios and relevant model parameters associated with CKD and ESRD according to baseline eGFR of 34.9mL/min/1.73m^2^ and ACR of 403 mg/g.

| Name | Description | Value | Standard Error | Source |
| --- | --- | --- | --- | --- |
| hr_CKD | CKD3/4 all-cause mortality hazard ratio | 5.05 | 0.137 | estimated |
| hr_CKD_f | CKD3/4 all-cause mortality hazard ratio - Females | 5.74 | 0.221 | (21) |
| hr_CKD_m | CKD3/4 all-cause mortality hazard ratio - Male | 4.46 | 0.179 | (21) |
| hr_ESRD | ESRD/CKD5 all-cause mortality hazard ratio | 13.17 | 0.157 | estimated |
| hr_ESRD_f | ESRD/CKD5 all-cause mortality hazard ratio - Female | 16.07 | 0.22 | (21) |
| hr_ESRD_m | ESRD/CKD5 all-cause mortality hazard ratio - Male | 11.64 | 0.156 | (21) |
| hr_ESRD1 | ESRD1 (dialysis) all-cause mortality ratio  (combined estimate for peritoneal- and haemodialysis) | 10.18 | 2.09 | (22) |
| hr_ESRD2 | ESRD2 (transplant) all-cause mortality ratio | 5.6 | 1.378 | (22) |

#### MSC Therapy Arm

The MSC arm was developed by a multidisciplinary group with specialised knowledge of the expected cost and effect of MSC therapy (www.nephstrom.eu). This involved a once-off treatment cost of approximately EUR€2,500/dose (SE: +/- 20%) incurred when a patient is in the CKD 3/4 stage of the model (S7 Table). The future cost of MSC therapy is uncertain but is likely to be a function of the required dosage and/or economies of scale during production. Based on current manufacturing requirements and market value considerations, a cost of €2,500/dose was used (Personal communication, Dr. Stephen Elliman, Orbsen Therapeutics Ltd.).

The treatment effect was estimated using published preliminary efficacy data on the use of allogeneic mesenchymal precursor cells – a MSC-like cell product - in DKD (4). Using approximate estimates for eGFR (and 95% Confidence Intervals) reported at baseline (34.9 mL/min/1.73m^2^; SE: 1.9) and changes in baseline eGFR every 12 weeks for 60 weeks (estimated to within 0.25mL/min/1.73 m^2^ from figure 3), a sample of 100,000 individuals for each treatment arm (150M and 300M) and placebo according to eGFR decline at each 12-week period post-baseline was simulated using a normal distribution. This provides an estimate of the HR associated with MSC therapy (0.83) and a SE of 20% of the HR (24) was used for PSA. Estimated eGFR at each window of measurement was categorised according to CKD stage and survival analysis was conducted using Cox regression to estimate the HR of entering ESRD/CKD 5 (eGFR < 15mL/min/1.73m^2^) for the combined treatment arms (150M and 300M) compared to the placebo. Individuals receive the benefits of MSC therapy while in the CKD 3/4 stage which ceases upon entry to ESRD/CKD5.

S7 Table: Hazard ratios for the reduced likelihood of transitioning to ESRD from MSC and treatment cost (€2,500) converted to USD 2019.

| Name | Description | Value | Standard Error | Source |
| --- | --- | --- | --- | --- |
| c_MSC | MSC cost - 1 dose (€2,500) | $3,278 | +/- 20% | S.Elliman, pers com |
| hr_MSC | MSC risk reduction | 0.83 | +/- 20% | (4), Simulated |

#### SGLT2i Therapy Arm

SGLT2i class effects were estimated in a systematic review and meta-analysis for the reduced risk of ESRD and death relative to UC and are outlined in S8 Table below (25). Estimates of the monthly cost of SGLT2i therapy were based on the arithmetic mean of a monthly prescription for canagliflozin (100mg), dapagliflozin (10mg) and empagliflozin (10mg) using drug price lists separately for Ireland (26), UK (27) and Italy (28). Individuals receive the benefits and costs of SGLT2i while in CKD with treatment ceasing upon entry to ESRD/CKD5 and no subsequent treatment effect.

S8 Table: Hazard ratios for the reduced likelihood of transitioning to ESRD or death from SGLT2i therapy and monthly prescription costs per country converted to USD 2019

| Name | Description | Value | Standard Error | Source |
| --- | --- | --- | --- | --- |
| c_SGLT2i_Ire | Monthly SGLT2i prescription cost - Ireland | 53.32 | 0.879 | (26) |
| c_SGLT2i_Ita | Monthly SGLT2i prescription cost - Italy | 90.83 | 5.269 |  |
| c_SGLT2i_UK | Monthly SGLT2i prescription cost - UK | 57.03 | 0.006 | (27) |
| c_SGLT2i_avg | Monthly SGLT2i prescription cost - average | 67.06 | 18.1 | estimated |
| hr_SGLT2i_D | Hazard ratio for SGLT2i all-cause mortality | 0.77 | 95% CI: 0.71 – 0.83 | (25) |
| hr_SGLT2i_E | Hazard ratio for SGLT2i kidney failure/ESRD/CKD5 | 0.71 | 95% CI: 0.57 – 0.89 | (25) |

#### Model Validation

To account for the progressive nature of DKD over time, a Markov jump process was included such that HRs for transition to ESRD or death increase in proportion to the number of cycles in the model (29). We use external data on time to ESRD or death to configure the speed with which progression of disease increases in the model. The jump process is structured such that HRs are multiplied by a factor proportionate to the stage of the model, with a factor of ‘1’ reflecting a scenario where HRs will double every year and a factor of ’10’ reflecting a scenario whereby HR’s will double every 10 years.

It is expected that a man age 40 years in CKD 3 has a remaining life expectancy of 24 years and a corresponding 28 years for a woman (30). While another study of patients in CKD 3 found that approximately 50% progressed to advance stages of kidney disease after 10 years (31). Inputting the baseline characteristics for men/women from this study, an age of 40 in CKD 3 and ACR approximately 210 mg/g in UC in the model, adjustment of the jump process was used to validate the model. A jump of ‘4’ (HRs doubling every 4 years) predicted that 50% of individuals moved from CKD to ESRD after c.10 years (122 months) and 50% of individuals in the model died after 26.6 years (319 months) and was thus used as the jump process in the model.

## References

1. Sugrue DM, Ward T, Rai S, McEwan P, van Haalen HGM. Economic Modelling of Chronic Kidney Disease: A Systematic Literature Review to Inform Conceptual Model Design. PharmacoEconomics 2019;37:1451-1468

2. Tangri N, Grams ME, Levey AS, Coresh J, Appel LJ, Astor BC, Chodick G, Collins AJ, Djurdjev O, Elley CR. Multinational assessment of accuracy of equations for predicting risk of kidney failure: a meta-analysis. Jama 2016;315:164-174

3. Tangri N, Stevens LA, Griffith J, Tighiouart H, Djurdjev O, Naimark D, Levin A, Levey AS. A Predictive Model for Progression of Chronic Kidney Disease to Kidney Failure. JAMA 2011;305:1553-1559

4. Packham DK, Fraser IR, Kerr PG, Segal KR. Allogeneic Mesenchymal Precursor Cells (MPC) in Diabetic Nephropathy: A Randomized, Placebo-controlled, Dose Escalation Study. EBioMedicine 2016;12:263-269

5. Gray AM, Clarke PM, Wolstenholme JL, Wordsworth S. *Applied methods of cost-effectiveness analysis in healthcare*. Oxford University Press, 2010

6. Walter S, Yao X. Effect sizes can be calculated for studies reporting ranges for outcome variables in systematic reviews. Journal of clinical epidemiology 2007;60:849-852

7. de Vries EF, Rabelink TJ, van den Hout WB. Modelling the Cost-Effectiveness of Delaying End-Stage Renal Disease. Nephron 2016;133:89-97

8. Li B, Cairns JA, Draper H, Dudley C, Forsythe JL, Johnson RJ, Metcalfe W, Oniscu GC, Ravanan R, Robb ML, Roderick P, Tomson CR, Watson CJE, Bradley JA. Estimating Health-State Utility Values in Kidney Transplant Recipients and Waiting-List Patients Using the EQ-5D-5L. Value in Health 2017;20:976-984

9. StataCorp. Stata Statistical Software: Release 16. 16 ed. College Station, TX, StataCorp LLC, College Station, TX, 2019

10. Higgins JPT, Li T, Deeks JJ. Chapter 6: Choosing effect measures and computing estimates of effect. In *Cochrane Handbook for Systematic Reviews of Interventions version 61,* 6.1 ed. Higgins JPT, Thomas J, Chandler J, Cumpston M, Li T, Page MJ, Welch VA, Eds., Cochrane, 2020

11. Elshahat S, Cockwell P, Maxwell AP, Griffin M, O’Brien T, O’Neill C. The impact of chronic kidney disease on developed countries from a health economics perspective: A systematic scoping review. PLOS ONE 2020;15:e0230512

12. Kerr M. Chronic kidney disease in England: the human and financial cost. NHS Kidney Care 2012;

13. Tajima R, Kondo M, Kai H, Saito C, Okada M, Takahashi H, Doi M, Tsuruoka S, Yamagata K. Measurement of health-related quality of life in patients with chronic kidney disease in Japan with EuroQol (EQ-5D). Clinical and experimental nephrology 2010;14:340-348

14. NICE technology appraisal guidance [article online], 2019. Available from <https://www.nice.org.uk/About/What-we-do/Our-Programmes/NICE-guidance/NICE-technology-appraisal-guidance>. Accessed October 2019

15. Health Information and Quality Authority. Guidelines for the Economic Evaluation of Health Technologies in Ireland 2018. 2018

16. Labour force participation rate (indicator) [article online], 2020. Available from <https://data.oecd.org/emp/labour-force-participation-rate.htm>. Accessed October 21st 2020

17. Average wages (indicator) [article online], 2020. Available from <https://data.oecd.org/earnwage/average-wages.htm>. Accessed October 21st 2020

18. Jansen DL, Rijken M, Heijmans MJWM, Kaptein AA, Groenewegen PP. Psychological and Social Aspects of Living with Chronic Kidney Disease. 2012;

19. Reilly MC, Zbrozek AS, Dukes EM. The Validity and Reproducibility of a Work Productivity and Activity Impairment Instrument. PharmacoEconomics 1993;4:353-365

20. Eriksson D, Karlsson L, Eklund O, Dieperink H, Honkanen E, Melin J, Selvig K, Lundberg J. Real-world costs of autosomal dominant polycystic kidney disease in the Nordics. BMC Health Serv Res 2017;17:560

21. Nitsch D, Grams M, Sang Y, Black C, Cirillo M, Djurdjev O, Iseki K, Jassal SK, Kimm H, Kronenberg F, Oien CM, Levey AS, Levin A, Woodward M, Hemmelgarn BR, Chronic Kidney Disease Prognosis C. Associations of estimated glomerular filtration rate and albuminuria with mortality and renal failure by sex: a meta-analysis. BMJ (Clinical research ed) 2013;346:f324-f324

22. Neovius M, Jacobson SH, Eriksson JK, Elinder C-G, Hylander B. Mortality in chronic kidney disease and renal replacement therapy: a population-based cohort study. BMJ Open 2014;4:e004251

23. Human Mortality Database [article online], Available from [www.mortality.org](file:///Users/lukebarry_1/Dropbox/Research/Projects/DKD%20Project/Publication/Submission/PLoS%20One/Submission/Resubmission/www.mortality.org). Accessed October 21st 2020

24. Health Information and Quality Authority. Guidelines for the Economic Evaluation of Health Technologies in Ireland 2020. 2020

25. Palmer SC, Tendal B, Mustafa RA, Vandvik PO, Li S, Hao Q, Tunnicliffe D, Ruospo M, Natale P, Saglimbene V, Nicolucci A, Johnson DW, Tonelli M, Rossi MC, Badve SV, Cho Y, Nadeau-Fredette A-C, Burke M, Faruque LI, Lloyd A, Ahmad N, Liu Y, Tiv S, Millard T, Gagliardi L, Kolanu N, Barmanray RD, McMorrow R, Raygoza Cortez AK, White H, Chen X, Zhou X, Liu J, Rodríguez AF, González-Colmenero AD, Wang Y, Li L, Sutanto S, Solis RC, Díaz González-Colmenero F, Rodriguez-Gutierrez R, Walsh M, Guyatt G, Strippoli GFM. Sodium-glucose cotransporter protein-2 (SGLT-2) inhibitors and glucagon-like peptide-1 (GLP-1) receptor agonists for type 2 diabetes: systematic review and network meta-analysis of randomised controlled trials. BMJ 2021;372:m4573

26. Primary Care Reimbursement Service [article online], 2020. Available from <https://www.sspcrs.ie/druglist/pub>. Accessed 12th December 2020

27. British National Formulary [article online], 2021. Available from <https://www.nice.org.uk/bnf-uk-only>. Accessed 12th December 2020

28. Prices and Refunds [article online], 2021. Available from <https://www.aifa.gov.it/prezzi-e-rimborso>. Accessed March 2021

29. Briggs A, Sculpher M, Claxton K. *Decision modelling for health economic evaluation*. OUP Oxford, 2006

30. Neild GH. Life expectancy with chronic kidney disease: an educational review. Pediatr Nephrol 2017;32:243-248

31. Baek SD, Baek CH, Kim JS, Kim SM, Kim JH, Kim SB. Does stage III chronic kidney disease always progress to end-stage renal disease? A ten-year follow-up study. Scandinavian journal of urology and nephrology 2012;46:232-238
